# Convenient Biochemical Testing Technologies for Oral Disease Risk Warning: Opportunities and Challenges

**DOI:** 10.3390/bios15050327

**Published:** 2025-05-20

**Authors:** Ying Liu, Jincheng Xu, Siyuan Wang, Yuanfang Li, Li Ji, Dong Xie, Jianhua Zhou

**Affiliations:** 1 Key Laboratory of Sensing Technology and Biomedical Instruments of Guangdong Province, School of Biomedical Engineering, Shenzhen Campus of Sun Yat-sen University, Shenzhen 518107, China; liuyng6@mail2.sysu.edu.cn (Y.L.); xujch25@mail2.sysu.edu.cn (J.X.); wangsy88@mail2.sysu.edu.cn (S.W.);; 2 Guangdong Biomaterials Engineering Technology Research Center, Institute of Biological and Medical Engineering, Guangdong Academy of Sciences, Guangzhou 510316, China

**Keywords:** biochemical testing, oral diseases, dentistry, imaging, diagnosis, wearables

## Abstract

In recent years, attention toward oral health issues has increased with economic development and improvements in quality of life. Biochemical testing technologies offer an efficient method for identifying insidious pathological changes in the oral cavity. Frequent home-based self-screening can enable early identification of dental disease risks, thus facilitating timely interventions. Convenient home-based biochemical testing methods must be user-friendly, cost-effective, and operable without specialized equipment or extensive training. This review summarizes recent advances in convenient biochemical testing methods for the detection and diagnosis of oral diseases, focusing on their reliability, user compliance, and practicality for home-based applications. This review highlights the significance of biomarker distribution imaging for simultaneously identifying multiple lesions and provides perspectives on future research directions. By promoting interdisciplinary collaboration in biochemical diagnostics, this review outlines pathways toward personalized oral healthcare, precision dentistry, and enhanced overall health outcomes.

## 1. Introduction

As the initial segment of the digestive tract, the oral cavity serves as a primary barrier for overall health. In addition to performing physiological functions as food mastication and nutrient absorption, a healthy oral cavity significantly influences personal appearance, enhances self-confidence, and contributes positively to overall happiness and self-esteem. Oral health is closely associated with oral hygiene status. Poor oral hygiene creates an environment conducive to bacterial proliferation, and the excessive growth of bacteria can disrupt the local immune equilibrium, thereby leading to the development of oral diseases [1]. Oral diseases, including dental caries, periodontitis, and oral cancer, are among the most prevalent health issues worldwide [2]. In 2022, approximately 3.5 billion individuals were affected by oral diseases, accounting for nearly 45% of the global population. This figure exceeds the combined total number of patients suffering from cardiovascular diseases, diabetes, chronic respiratory diseases, and cancers by about one billion [3]. The economic burden of oral diseases is also staggering, with direct treatment costs and productivity losses associated with these conditions reaching an estimated USD 710 billion annually [3]. Furthermore, oral diseases are strongly associated with a variety of systemic health conditions [4]. The bacteria present around untreated oral lesions can infiltrate into the bloodstream through surrounding soft tissues and small blood vessels, triggering systemic inflammation and immune responses [5,6]. This translocation of pathogens can contribute to the development or exacerbation of infectious diseases [7], cancer [8], metabolic disorders [9], and cardiovascular diseases [10], posing a serious and multifaceted threat to both physical and mental health.

Oral diseases can often be prevented from progressing into more severe stages through early diagnosis and intervention. For instance, dental caries and periodontal disease, which are among the most common oral diseases, can be reversed with early diagnosis and prompt treatment, preventing more serious outcomes like pulpitis, periodontitis, gum recession, and tooth loss [11]. However, due to the hidden symptoms of oral diseases, many patients fail to pursue prompt care, allowing the diseases to progress into chronic conditions that require long-term treatment. This oversight not only worsens oral health but also increases the risk of systemic diseases, highlighting the urgent need for the early diagnosis of oral diseases.

To detect and prevent oral disease, dental experts recommend several practices. First, individuals should maintain good oral hygiene by brushing their teeth twice daily and flossing regularly. Additionally, experts advise undergoing oral examinations regularly [12,13]. These examinations typically include clinical evaluation and imaging tests (e.g., X-rays and computed tomography (CT)) [14,15]. However, current oral examination methods have several limitations: (1) There are low attendance rates among patients with mild symptoms. Clinic visits are often inconvenient, time-consuming, and costly. As a result, individuals frequently overlook mild pain, bleeding, and swelling associated with early oral diseases [16,17]. This leads to delayed diagnosis and treatment. (2) Lesions at the early stage with mild symptoms are easily missed. The complexity of oral structures often conceals early lesions, leading to missed diagnoses during inspections and palpation. (3) The uneven distribution of medical resources. In economically middle-income countries, there is a shortage of experienced dentists and advanced imaging equipment, limiting access to essential oral health services. These factors have contributed to the rising incidence of oral diseases. Over the past two decades, the global prevalence of oral diseases has increased by nearly one billion, reflecting a 50% rise [3]. These statistics highlight the inadequacy of existing oral healthcare systems, underscoring the urgent need for advanced oral disease warning technologies.

Biochemical detection technology is an important tool in modern medicine. It measures specific biomarkers (e.g., cytokines, antibodies, inflammatory mediators, and bacterial metabolites) in body fluids (e.g., blood [18], saliva [19], and sweat [20]) to reflect changes in the body’s health status. Given that oral diseases involve biochemical processes like bacterial proliferation, immune activation, and inflammation, biochemical detection plays an important role in the early screening and diagnosis of oral disease risks [21]. Convenient and frequent at-home detection can facilitate the early warning of dental disease risks, thereby enabling timely interventions and significantly improving oral health outcomes. Recent advancements in convenient, non-invasive biochemical testing technologies, such as breath analysis, saliva testing, and wearable devices, along with the Internet of Things (IoT) and artificial intelligence (AI), have made home-based oral disease screening increasingly feasible [22].

However, for at-home biochemical screening methods to achieve widespread adoption, they must be convenient, easy to use, affordable, accessible, and suitable for frequent use at home without requiring specialized equipment or skilled operation. This definition serves as a key criterion in evaluating biochemical testing methods, highlighting the importance of user-friendly designs, cost-effectiveness, and practical operability in daily home settings.

**Scheme 1:**
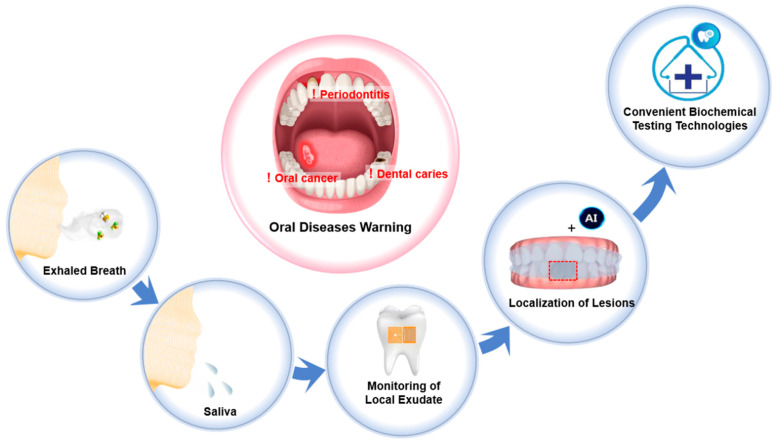
The current status and future prospects of convenient biochemical testing technologies for oral disease risk warning. The red dashed box highlights the localized lesion site. Reproduced with permission [23]. Copyright 2022, Elsevier. Reproduced with permission [24]. Copyright 2019, American Chemical Society. Reproduced with permission [25]. Copyright 2012, Springer Nature. Reproduced with permission [26]. Copyright 2022, Wiley-VCH.

This review introduces the pathological processes and biomarkers associated with oral diseases at the early stage (the Introduction of Section 2). Then, it highlights recent advancements in biochemical detection technologies for oral disease risk warning, focusing on the challenges related to sample sourcing. Furthermore, it discusses the potential and challenges of these technologies regarding convenience and accuracy in practical applications (Section 2.1, Section 2.2, Section 2.3 and Section 2.4). In summary, a comparative table presenting key performance indicators of the discussed technologies is provided. In the Section 3, this review emphasizes the scattered and insidious nature of oral disease pathologies, highlighting the significance of biomarker distribution imaging for simultaneously identifying multiple lesions. Finally, future directions and opportunities for convenient biochemical detection in oral health management are discussed (Section 4). Scheme 1 illustrates the current status and future prospects of convenient biochemical testing technologies for oral disease risk warning.

## 2. Biochemical Testing Technologies for Oral Diseases Warning

This review examines the relevant literature based on three key factors that need to be considered when developing a convenient biochemical testing method for early oral diseases warning: (1) biomarkers and their sources must strongly correlate with the pathological processes of oral diseases; (2) detection technologies should offer adequate specificity, sensitivity, and an appropriate concentration range for clinical use; and (3) test results must be easily interpretable without requiring specialized equipment. To begin with, the biomarkers of oral diseases at the early stage are discussed. Biomarkers, as defined by the WHO [27], are substances, structures, or processes that can be measured in the body or its products and influence or predict the occurrence of an outcome or disease. Based on this definition, Table 1 summarizes the lesion locations, pathological processes, and corresponding biomarkers of major oral diseases at the early stage.

It should be noted that samples from different sources contain biomarkers released from specific regions of the body. For example, exhaled breath contains biomarkers from the lungs, esophagus, and oral cavity, while saliva contains biomarkers from the salivary glands and local exudate. However, if a biomarker can be released from multiple regions and accumulates in the same sample, it becomes challenging to attribute the biochemical detection results to a specific site of disease. Since biomarkers from different sources vary in their correlation with oral disease pathology, the following discussion categorizes biochemical detection technologies based on the sources of the samples.

### 2.1. Measuring Biomarkers in Exhaled Breath

Exhaled breath offers a promising non-invasive source of biomarkers for detecting dental and oral mucosal diseases, enabling rapid screening and early diagnosis. Compared with traditional blood tests, breath collection is straightforward, making it ideal for continuous monitoring and real-time diagnosis [53]. Current methods for detecting biomarkers from exhaled breath include gas chromatography, portable respiratory devices (e.g., breathalyzers and carbon monoxide meters), and nanomaterial-based gas sensors. Gas chromatography, a standard laboratory technique, detects a broad range of trace compounds. However, the equipment is costly, bulky, and complex to operate. Portable devices using wet chemical methods are compact, sensitive, and fast, but require regular calibration and maintenance.

**Figure 1:**
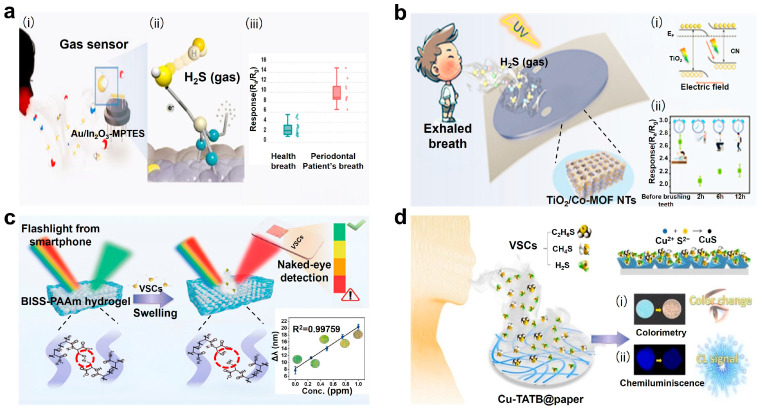
Detection of volatile sulfur compounds (VSCs) in exhaled breath for the warning of periodontitis. (**a**) Chemiresistor for the detection of exhaled H_2_S using an Au/In_2_O_3_-MPTES gas sensor. (i) Schematic of the sensing process; (ii) MPTES enhances the sensitivity of Au/In_2_O_3_ to H_2_S. (iii) Detection results of exhaled breath from healthy volunteers and periodontitis patients. Reproduced with permission [58]. Copyright 2022, American Chemical Society. (**b**) Chemiresistor for the detection of exhaled H_2_S using a TiO_2_/Co-MOF Z-Scheme heterojunction gas sensor at room temperature. (i) Z-scheme heterojunctions enhance the sensitivity of TiO_2_/Co-MOF to H_2_S by providing abundant photogenerated electrons. (ii) Detection results of exhaled breath from a volunteer. Reproduced with permission [59]. Copyright 2023, American Chemical Society. (**c**) Colorimetric detection of VSCs using a BISS-PAAm hydrogel. The red dashed circle highlights the changes in disulfide bonds before and after the reaction. Reproduced with permission [60]. Copyright 2024, Royal Society of Chemistry. (**d**) Dual-mode detection of exhaled VSCs using a copper metal–organic framework (Cu-MOF) paper-based sensor. (i) Colorimetry; (ii) chemiluminescence. Reproduced with permission [23]. Copyright 2022, Elsevier.

Recently, nanomaterial-based gas sensors, especially those based on semiconductor metal oxides (SMOs), have gained attention due to their high sensitivity, rapid response, stability, and potential for integration into wearable devices [54,55]. These sensors detect biomarkers through oxygen anion-mediated modulation of electrical properties [56]. In the air, oxygen molecules adsorb onto the surfaces of SMOs and capture free electrons from the conduction band, forming chemisorbed oxygen anions (O_2_^−^). When biomarker molecules interact with these chemisorbed O_2_^−^ on the surfaces of the SMOs, redox reactions occur, altering either the SMOs’ resistance (via electron transfer between O_2_^−^ and the conduction band) or capacitance (via modification of the space charge layer or dielectric properties). This mechanism directly links the biomarker concentration to measurable electrical signals [57]. Representative technologies for exhaled breath detection indicating oral disease risks are summarized in Figure 1.

Volatile sulfur compounds (VSCs), including hydrogen sulfide (H_2_S), methyl mercaptan (CH_3_SH), and dimethyl sulfide (CH_3_SCH_3_), are produced by anaerobic bacteria, such as *Porphyromonas gingivalis*, *Fusobacterium nucleatum*, and *Treponema denticola*, in periodontal plaque [61,62,63]. These anaerobic bacteria synthesize multiple enzymes that are involved in H_2_S production [64], thus making H_2_S the predominant VSC in the profiles of periodontitis patients [35]. Recently, the development of SMOs for detecting exhaled VSCs or H_2_S has become a key focus for non-invasive periodontitis screening, yielding significant advancements. To enhance the selectivity of the sensors, Zhang et al. [58] developed a self-assembled monolayer (SAM)-functionalized Au/In_2_O_3_ nanofiber sensor (Figure 1a(i)). By adjusting the terminal groups and alkyl chains of the SAMs, stronger interactions between MPTES and H_2_S were achieved, improving the sensor’s binding capacity to H_2_S (Figure 1a(ii)). As shown in Figure 1a(iii), the Au/In_2_O_3_-MPTES sensor, which measures resistance changes, successfully distinguishes the breath of healthy individuals from that of patients with severe periodontitis. This sensor exhibits a detection limit of 10 ppb and a selectivity 145 times greater for H_2_S at 0.1 ppm compared with five other interfering gases at 100 ppm. However, the sensor must be operated at 100 °C, presenting a challenge for monitoring exhaled breath directly.

To solve the problem of the high operating temperature, Zhao et al. [59] proposed a novel strategy by using ultraviolet light (365 nm, Futansi, Beijing, China) to excite the TiO_2_/Co-MOF nanotubes with a Z-scheme heterojunction (Figure 1b). The photogenerated electrons from these Z-scheme heterojunctions enhance the sensor’s sensitivity, enabling the effective detection of H_2_S at room temperature (Figure 1b(i)). As shown in Figure 1b(ii), the sensor successfully monitors the concentration levels of H_2_S in a volunteer at different times of the day by detecting resistance changes. With a detection limit of 1.3 ppb, the sensor shows excellent selectivity, even against 100 times higher concentrations of interference gases. Also, Chen et al. [65] proposed a Cu_2_O/ZnO heterojunction-based H_2_S gas sensor that operates at temperatures as low as −20 °C to room temperature. The sensor achieved a detection limit as low as 10 ppb at room temperature and demonstrated exceptional sensitivity, with the response to 50 ppm H_2_S being 3774 times greater than that of a CuZnO sensor.

The high humidity in the oral cavity (85~96%) also poses a significant challenge for most metal oxide gas sensors. The surface coverage and reactivity of O_2_^−^ are not only crucial for generating electrical signals but also directly influence sensor performance [66]. Water molecules alter the distribution of chemisorbed O_2_^−^ on the surface of SMOs through competitive adsorption, thereby interfering with the resistance baseline (causing conductivity drift) or capacitance response (leading to dielectric property distortion), which subsequently reduces the sensor’s sensitivity [67]. To overcome this, Takura et al. [68] introduced a layered manganese oxide nanosheet coupled with a quartz crystal microbalance sensor. This sensor shows improved responsiveness to CH_3_SH under humid conditions, with a detection limit of 20 ppb. Du et al. [69] reported a Fe-doped MoO_3−x_ TiO_2_ nanotube-based chemiresistive H_2_S sensor. This sensor retained over 95% of its initial response under 70% relative humidity, and achieved a detection limit down to 0.34 ppb, ranking it among one of the most sensitive room-temperature chemiresistive H_2_S sensors reported to date. These findings provide valuable insights for developing exhaled-breath sensors in high-humidity oral environments.

To facilitate result visualization, Hu et al. [60] developed a colorimetric detection method for VSCs using a BISS-PAAm hydrogel (Figure 1c). VSCs reduced the disulfide bonds in the hydrogel network, causing expansion and a color change. This method offers a linear detection range of 0~1 ppm and a detection limit of 61 ppb. Furthermore, Hou et al. [23] developed a dual-modal colorimetric and chemiluminescent detection method for VSCs by using a Cu-TATB@paper sensor (Figure 1d). The colorimetric method has a detection limit of 0.2 μM, while the chemiluminescent method achieves a detection limit of 8 nM. These advancements provide innovative approaches for the convenient biochemical detection of oral diseases.

Although exhaled breath analysis is non-invasive and convenient, its application to oral disease screening remains limited by (1) the insufficient specificity of biomarkers (e.g., H_2_S may originate from respiratory diseases [70], halitosis [71], and colorectal cancer [72]); (2) pronounced biological variability (inter-individual physiological differences and sampling parameters, such as flow rate, duration, and nasal versus oral breathing [73]); and (3) environmental interference (ambient humidity’s effects on sensor performance and cross-reactivity among analytes). Therefore, it is imperative to develop a standardized breath-sampling protocol and to optimize the specificity of sensing materials in order to mitigate these factors and improve the reliability of oral disease screening.

### 2.2. Detecting Biomarkers in Whole Saliva

**Figure 2:**
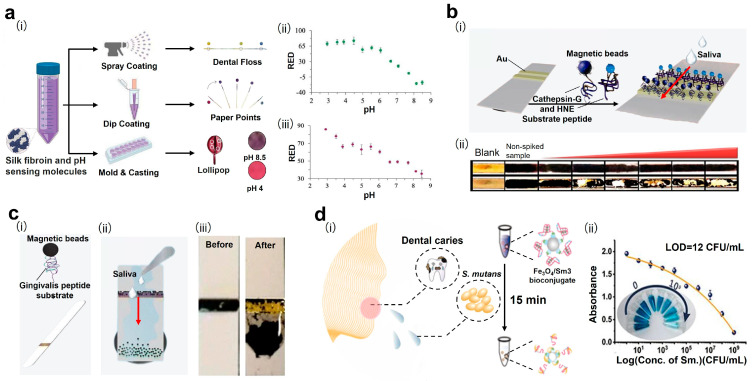
Detection of pH, proteases, and bacteria in saliva for oral disease warning. (**a**) Colorimetric detection of pH in oral cavity by pH-sensing molecule-functionalized interfaces for the warning of dental caries. (i) Schematic of the development of pH-sensing dental floss, paper points, and lollipops. (ii) Sensing response of Nitrazine yellow-based dental floss at different pH values. (iii) Sensing response of anthocyanins-based paper points at different pH values. Reproduced with permission [79]. Copyright 2021, Wiley-VCH. (**b**) Colorimetric detection of human neutrophil elastase (HNE) and cathepsin-G in saliva using a magnetic nanobead-based assay for the warning of periodontitis. The red arrow indicates the flow direction of saliva. (i) Schematic of the preparation and detection process. (ii) Detection results for saliva samples from patients. Reproduced with permission [80]. Copyright 2015, American Chemical Society. (**c**) Colorimetric detection of gingipain protease in saliva using a magnetic nanobead-based assay for the warning of periodontitis. The red arrow indicates the flow direction of saliva. Schematic of the (i) preparation and (ii) detection process of the assay. (iii) Detection results for saliva samples from patients. Reproduced with permission [81]. Copyright 2018, Springer Nature. (**d**) Colorimetric detection of *Streptococcus mutans* (*S. mutans*) in saliva using engineered DNA–nanozyme interfaces for the warning of dental caries. (i) Schematic of the preparation and detection process. (ii) Detection results for different samples of *S. mutans*. Reproduced with permission [24]. Copyright 2019, American Chemical Society.

Whole saliva in the oral cavity is composed of saliva secreted from the major and minor salivary glands, along with non-salivary fluids, such as nasal drip and gingival crevicular fluid (GCF) [74]. Compared with blood collection, whole-saliva sampling is non-invasive, significantly improving patient compliance. As research advances on the high correlation between the biochemical content in saliva and blood, whole-saliva detection has become an important method for monitoring various physiological parameters, including emotional state, hormone levels, nutritional status, and metabolic activities [75]. Notably, oral disease biomarkers originate specifically from oral lesions and appear directly in whole saliva. Thus, they provide more localized insights into oral health conditions [76,77]. To detect these biomarkers, various biochemical detection technologies have been developed, including colorimetry, fluorescence methods, immunochromatography, polymerase chain reaction (PCR), electrochemical techniques, and piezoelectric methods [78]. Among these, colorimetry, fluorescence techniques, and immunochromatography are commonly used for point-of-care testing and on-site analysis due to their rapid response, cost-effectiveness, minimal instrumentation requirements, and compatibility with non-laboratory settings. Representative technologies for whole saliva detection indicating oral disease risks are summarized in Figure 2.

Numerous studies have indicated that when the body is infected by bacteria or experiences an autoimmune disorder causing oral diseases, local tissue will release various substances produced by bacterial metabolism and immune responses. These substances include organic acids, VSCs, chemokines, inflammatory factors, enzymes, and the bacteria themselves [82,83]. For instance, when cariogenic bacteria like *Streptococcus mutans* (*S. mutans*) and *Lactobacillus* metabolize food-derived carbohydrates, acids are produced, lowering the pH on the enamel surface. When the pH drops to around 5.5, enamel minerals will start to dissolve, initiating the process of dental caries [84]. Therefore, local pH levels in the oral cavity can serve as an indicator of the caries risk. Anthocyanins, one of the natural pigments that are safe and pH-sensitive, offer a promising approach for pH detection. As shown in Figure 2a(i), Matzeu et al. [79] embedded anthocyanins into silk fibroin to develop edible pH sensors, such as dental floss, toothpicks, and candies. This work provides a convenient at-home solution for oral pH detection.

For detecting biological macromolecules like nucleic acids, proteins, and enzymes, gold nanoparticles (AuNPs) are widely used [85]. AuNPs modified with specific DNA sequences or antibodies can rapidly identify macromolecular biomarkers of oral diseases, achieving color change results through the localized surface plasmon resonance (LSPR) characteristics of gold nanoparticles. For instance, the saliva-check mutans kit [86], developed by GC Company in Japan, utilizes AuNP-based immunochromatography to detect *S. mutans* in saliva in just 15 min, providing an effective screening tool for dental caries. In addition, recombinase polymerase amplification (RPA) technology overcomes the limitations of traditional PCR, which requires high-temperature cycles for DNA amplification. As a result, it improves DNA detection by making it faster, simpler, and portable. Jiang et al. [87] integrated RPA technology with lateral-flow (LF) strips and achieved a detection limit of 6.40 × 10^−4^ μg/mL for *Porphyromonas gingivalis* DNA. This assay is rapid (result comes out within 15 min) and easy to perform, although it still requires DNA extraction prior to detection.

Given the complexity of saliva and the low concentrations of early-stage biomarkers, surface-modified magnetic nanoparticles (MNPs) offer a promising solution [88]. These nanoparticles specifically recognize and capture the target molecules of biomarkers and can then be separated rapidly using an external magnetic field, improving the sensitivity of biomarker detection in saliva. For example, Wignarajah et al. [80] developed a colorimetric sensor to detect human neutrophil elastase (HNE) and cathepsin-G, achieving detection limits of 1 pg/mL for HNE and 100 fg/mL for cathepsin-G (Figure 2b), respectively. When the saliva sample is added onto the sensor, HNE and cathepsin-G in the saliva induce proteolysis to their respective substrates, leading to the cleavage of the bonds between the magnetic beads and the gold sensor surface. The cleaved magnetic beads are then attracted by an external magnet, revealing the gold color of the sensor surface. Alhogail et al. [81] employed a similar approach to develop a sensor for gingipains by detecting *Porphyromonas gingivalis* at 49 CFU/mL in just 30 s (Figure 2c). To enhance MNPs’ efficiency in capturing specific bacteria from complex saliva samples, Zhang et al. [24] proposed a colorimetric sensing method for detecting *S. mutans* in saliva by developing MNPs modified with DNA aptamers to target *S. mutans* (Figure 2d), achieving a detection limit of 12 CFU/mL (Figure 2d(ii)), suggesting its potential in dental caries screening. Moreover, Nguyen et al. [89] reported a PMMA-based microfluidic chip for detecting odontogenic ameloblast-associated protein (ODAM) to diagnose periodontitis, achieving a detection limit of 0.011 nM for ODAM. However, this method heavily relies on specialized equipment and skilled operations, limiting its feasibility for home-based applications.

Oral cancer is a prevalent malignant tumor of the head and neck region. Studies indicate that human papillomavirus type 16 (HPV16) infection is a critical etiological factor in oral cancer [90,91]. Therefore, developing rapid, sensitive, and user-friendly methods for detecting HPV16 infection holds significant value for the early screening, risk assessment, and timely intervention of oral cancer.

Among current detection methods for HPV infection, identifying HPV-related protein biomarkers, such as viral antigens and host antibodies (e.g., HPV16 E6/E7 proteins and their respective antibodies), is a promising strategy [52]. However, due to the significantly lower concentrations of viral antigens and host antibodies in saliva [92] compared to serum, the existing assays lack sufficient sensitivity, making saliva-based antigen and antibody detection challenging for home-based screening applications.

In comparison, detection methods targeting viral DNA or RNA demonstrate greater potential for home-based screening. Recently, loop-mediated isothermal amplification (LAMP) and CRISPR/Cas technologies have shown notable progress in HPV16 gene detection. LAMP employs specific primers and enzymes, such as Bst, to rapidly amplify HPV16 DNA from saliva samples of patients with oral squamous cell carcinoma at a constant temperature of 60–65 °C. For instance, Hamzan et al. [93] developed a real-time fluorescent LAMP (qLAMP) assay targeting the HPV16 E7 gene, achieving a detection limit of 46.8 copies/µL, which represents a two-order magnitude improvement over conventional PCR methods. The CRISPR/Cas system integrates initial isothermal amplification techniques (such as RPA or LAMP) to amplify HPV16 nucleic acid fragments, followed by the recognition and cleavage of reporter probes by Cas12a or Cas13 enzymes guided by specific RNAs. Ghouneimy et al. [94] proposed an RPA-Cas12a combined method for detecting HPV16/18, achieving a theoretical detection limit as low as 10 copies/µL within approximately 40 min. This method demonstrates high specificity, compatibility with complex salivary microbiomes, and operation at moderate temperatures (37–42 °C), which minimizes dependency on specialized equipment, making it ideal for portable or at-home screening applications. While LAMP and CRISPR technologies offer promising potential for home-based oral cancer screening through HPV16 detection, it is crucial to acknowledge that a positive detection result only confirms viral presence and cannot distinguish between transient and persistent oncogenic infections. Thus, follow-up monitoring or combined analyses with additional molecular biomarkers, such as p16 protein or HPV mRNA, are essential for comprehensive risk assessments.

Although whole-saliva analysis is non-invasive and amenable to large-scale screening, its application to oral disease screening still faces several challenges: (1) It has a complex sample matrix and low analyte levels [95]. Saliva contains mucins, enzymes, and cellular debris that require centrifugation or filtration pretreatment, while target biomarkers are often present at extremely low concentrations, necessitating additional enrichment or concentration steps. (2) Its biological variability [96,97] means that inter-individual physiological differences, together with fluctuations in the sampling flow rate and duration, lead to significant result variability and reduced stability. Consequently, it is imperative to establish standardized saliva-sampling protocols and to develop highly sensitive, specific sensing materials that function without pretreatment, thereby improving both the convenience and reliability of salivary diagnostics.

### 2.3. Monitoring Biomarkers in Local Exudate

**Figure 3:**
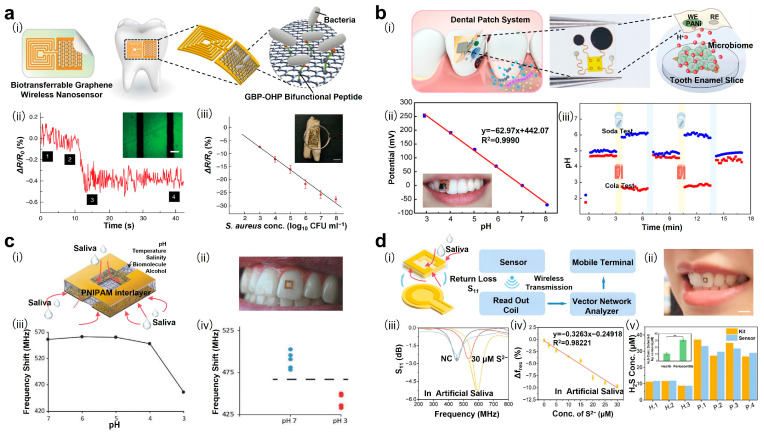
Detection of bacteria, pH, and H_2_S in localized secreted fluids for oral disease warning. (**a**) Graphene-based wireless electrical resistance sensor for the detection of bacteria on the surface of tooth enamel. (i) Schematic of the detection by the graphene nanosensor. (ii) Binding of a single *E. coli* bacterium onto the graphene nanosensor (stages 2 to 3). Inset shows the fluorescence image of the peptide-functionalized graphene surface (green), with the black regions representing electrodes. Scale bar is 250 μm. (iii) Percentage change in the resistance of graphene versus the concentration of *S. aureus*. Error bars indicate standard deviations (*n* = 3). Inset shows optical image of the graphene wireless sensor worn on the surface of a tooth. Scale bar is 1 cm. Reproduced with permission [25]. Copyright 2012, Springer Nature. (**b**) A wearable electrochemical potentiometric sensor for pH monitoring for the warning of dental caries. (i) Schematic of the dental patch sensor detecting H^+^ produced by oral microbial metabolism; (ii) linear curve of the potential response versus pH (*n* = 3). Error bars indicate standard deviations. Inset shows a volunteer wearing the flexible electrode array. (iii) Real-time monitoring of oral pH when volunteer drank pure water (pH = 6.8), acidic cola (pH = 2.5), and alkaline soda water (pH = 8.0), in turn. Reproduced with permission [99]. Copyright 2022, Springer Nature. (**c**) Resonant frequency (RF) sensors of pH monitoring for the warning of dental caries. The red arrow indicates the flow direction of the localized exudate. (i) Schematic of the sensor. (ii) Optical image of a volunteer wearing the RF sensor; (iii) RF shifts of the sensor as a function of pH; (iv) RF shifts of the sensor as a function of repeated pH changes between 3 and 7. Reproduced with permission [100]. Copyright 2018, Wiley-VCH. (**d**) A hydrogel-based RF sensor for H_2_S monitoring for the warning of periodontitis. The red arrow indicates the flow direction of the localized exudate. (i) Schematic of the detection and wireless data transmission process. (ii) Optical image of a volunteer wearing an RF sensor. (iii) Detection results of negative control (NC) and 30 μM S^2−^ by the sensor. (iv) Calibration curve of normalized RF changes of the sensor for the detection of S^2−^ (*n* = 3). (v) Detection results of H_2_S from the health group (H.1–H.3) and periodontitis group (P.1–P.4). The inset displays the significant difference in the concentrations of saliva H_2_S between the healthy (*n* = 3) and the periodontitis (*n* = 4) groups (*p* < 0.01). Reproduced with permission [101]. Copyright 2024, Elsevier.

In contrast with methods detecting biomarkers in whole saliva, oral wearable patch sensors offer significant advancements by directly measuring local exudates from the specific sites of the oral cavity [98]. By analyzing biomarkers from the enamel and oral mucosa, this approach addresses challenges, such as buffering, dilution, and the dissipation of biomarkers in whole saliva. The localized origin of these samples minimizes interference from biomarkers produced elsewhere in the body. This enables more precise detection of local biochemical changes, enhancing the accuracy and effectiveness of oral disease warnings. Representative technologies for local exudate detection indicating oral disease risks are summarized in Figure 3.

As shown in Figure 3a(i), Mannoor et al. [25] developed an electrical resistance patch sensor for detecting bacteria on the surface of tooth enamel by modifying a self-assembled GBP-OHP bifunctional peptide on graphene. This sensor detects bacterial binding by measuring changes in resistance (Figure 3a(ii)), achieving the detection of individual bacteria on the local surface of the tooth, with a detection limit of 1 bacterium·μL^−1^ and a linear range of 10^3^ to 10^8^ CFU/mL (Figure 3a(iii)). Additionally, the sensor incorporates a resonant coil, eliminating the need for onboard power and external connections, thereby enabling the remote online monitoring of bacteria in saliva. As shown in Figure 3b(i), Shi et al. [99] proposed a patch sensor with a polyaniline (PANi)-modified electrode to monitor H^+^ ions released from the surface of tooth enamel. Through protonation and deprotonation reactions, PANi transitions between its emerald base and emerald salt states, resulting in changes in the electrode’s circuit potential. As shown in Figure 3b(ii), the sensor’s response is linear within a pH range of 3 to 8. The patch was tested for the real-time monitoring of pH changes on the tooth surface after a volunteer consumed pure water (pH = 6.8), acidic cola (pH = 2.5), and alkaline soda (pH = 8.0), demonstrating its rapid response, real-time monitoring, and reusability (Figure 3b(iii)). As illustrated in Figure 3c(i,ii), Peter Tseng et al. [100] developed a conformal radio frequency (RF) structure to monitor pH on the tooth surface. As shown in Figure 3c(iii), the sensor exhibits a significant change in the RF in the range of pH 3~4, enabling it to successfully differentiate signals at pH 7 and pH 3 (Figure 3c(iv)). Given that dental enamel is at risk of acid erosion when the local pH falls below 5.5, further optimization of the sensor’s response within the pH range of 4.0~5.5 is required.

As shown in Figure 3d(i,ii), Pan et al. [101] reported a hydrogel RF sensor for the in situ detection of H_2_S. The RF sensor is based on an agarose hydrogel–chlorhexidine matrix containing silver nanoparticles with a split-ring resonator. The detection of H_2_S is based on the variations in the RF caused by interactions between Ag NPs and sulfide ions (Figure 3d(iii)). The detection limit of this method is 1.2 μM. Notably, both RF sensors mentioned above are adhered to the tooth enamel and measure the biomarkers in the whole saliva of the oral cavity. As a result, the detection reflects the average level of H_2_S across the entire oral cavity, rather than offering insights into localized hidden lesions. Therefore, more sophisticated designs are needed to enhance the sensors’ ability to detect biomarkers from local exudates, enabling more targeted and precise detection of oral pathologies.

The emergence of patch-based electrochemical and resistive sensing technologies offers significant potential for convenient, continuous, and intelligent monitoring of biomarkers in local exudates, marking a transformative advancement in oral health management. However, current patch sensors are limited to assessing small areas of the enamel surface at a time, and the relative rigidity of circuit components may hinder patient compliance. There is a need for interdisciplinary collaboration to develop highly integrated and comfortable sensor arrays that are capable of achieving synchronous detections across the entire dentition.

### 2.4. Localizing Lesions Through Imaging Distribution of Biomarkers

**Figure 4:**
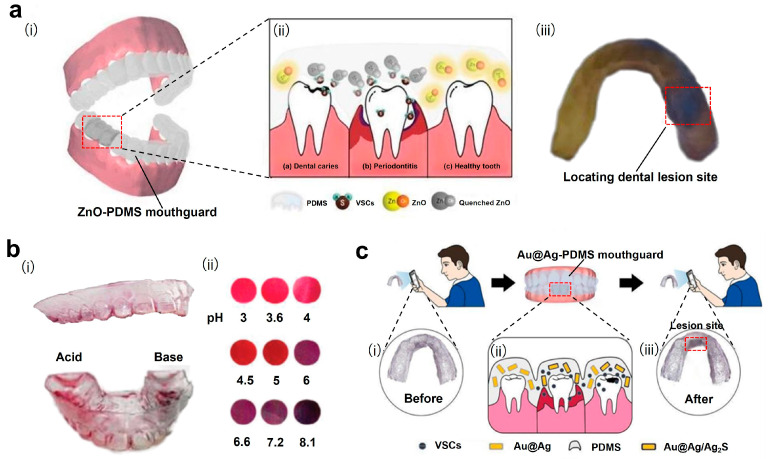
Imaging the location of oral lesions across the entire dentition by detecting H_2_S or H^+^ released from the whole dentition. (**a**) A fluorescent ZnO-PDMS mouthguard sensor for the detection of H_2_S to identify hidden periodontitis lesions. The red dashed box highlights the localized lesion site. (i) Schematic of the ZnO-PDMS mouthguard for lesion localizations. (ii) Local fluorescence quenching of the ZnO-PDMS mouthguard induced by H_2_S. (iii) Image of the ZnO-PDMS mouthguard after being worn, indicating hidden dental lesions. Reproduced with permission [102]. Copyright 2020, Wiley-VCH. (**b**) A colorimetric mouthguard coated with anthocyanins for pH detection to locate hidden lesions of dental caries. (i) Image of the pH-sensing mouthguard. (ii) Colorimetric response of the mouthguard recorded at different pH levels. Reproduced with permission [79]. Copyright 2021, Wiley-VCH. (**c**) Au@Ag nanorod-PDMS mouthguard sensor for colorimetric detection of H_2_S to locate hidden lesions of periodontitis. The red dashed box highlights the localized lesion site. (i) Schematic of Au@Ag nanorods-PDMS mouthguard before use. (ii) Local colorimetric change in the Au@Ag nanorod-PDMS mouthguard induced by H_2_S. (iii) Schematic of Au@Ag nanorod-PDMS mouthguard after use. Reproduced with permission [26]. Copyright 2022, Wiley-VCH.

In contrast, imaging the distribution of biomarkers can effectively identify and precisely locate lesions. These technologies enable the comprehensive screening of the entire dentition in a single detection session, offering clear advantages for oral disease warning and diagnosis. Representative technologies for lesions localization indicating oral disease risks are summarized in Figure 4.

As shown in Figure 4a(i), Li et al. [102] proposed a fluorescent ZnO-polydimethylsiloxane (PDMS) mouthguard sensor for the detection of H_2_S. This transparent ZnO-PDMS mouthguard, which exhibits fluorescence, responds to H_2_S (Figure 4a(ii)), resulting in an obvious quenching of fluorescence that enables the precise localization of periodontitis lesions (Figure 4a(iii)). The results were consistent with clinical diagnoses, demonstrating the potential of ZnO-PDMS mouthguard sensors for routine and convenient screening of oral diseases. As shown in Figure 4b(i), Matzeu et al. [79] proposed a colorimetric mouthguard sensor for imaging the pH levels on tooth surfaces. This sensor exhibits significant color changes in the pH range of 5 to 6 (Figure 4b(ii)), highlighting its potential for the routine screening of dental caries. As illustrated in Figure 4c(i,ii), Ma et al. [26] introduced a colorimetric Au@Ag-PDMS mouthguard sensor for H_2_S detection. After wearing this mouthguard for 7 h, the mouthguard exhibits a colorimetric response to H_2_S, enabling the localization of periodontitis lesions (Figure 4c(iii)). However, the prolonged wearing duration of ZnO-PDMS and the Au@Ag-PDMS mouthguard hinders user compliance. To address this, Liu et al. [103] proposed an ultra-thin dental patch by integrating ZnO quantum dots into a PDMS film and coupling it with AI recognition technology. This film sensor can accurately image the distribution of locally released H_2_S within 15 min, enabling the rapid screening and mapping of periodontal lesions. With a detection limit down to 20.3 µM for H_2_S, ZnO-PDMS dental patches show great promise for routine home-based periodontitis screening. Collectively, these studies highlight the promising future directions of convenient biochemical detection technologies for daily oral disease monitoring and risk assessment.

Fluorescence and colorimetric detection methods often suffer from insufficient sensitivity when detecting low concentrations of biomarkers. To overcome this limitation, artificial intelligence (AI) algorithms, particularly deep learning-based convolutional neural networks, can be employed to extract deeper features from images [104]. By utilizing supervised learning on datasets labeled with regions of interest (ROIs), AI models can segment images and identify areas where optical signals have changed, thereby facilitating the precise identification and screening of hidden lesions within the oral cavity [105]. The recent approval of two AI-based medical devices by the U.S. Food and Drug Administration (FDA) for radiological image analysis further indicates the feasibility of using AI to enhance diagnostic accuracy in periodontitis [106,107]. However, several technical challenges remain for applying AI in home-based biochemical diagnostics. First, smartphone-based colorimetric detection is susceptible to variations in lighting conditions, camera angles, and device models. Although standardized image acquisition protocols can mitigate these inconsistencies, such requirements often reduce user compliance. Second, training high-performance AI models requires extensive datasets labeled by medical professionals. Third, there is currently a lack of technical justification for diagnostic models, complicating the comparative assessment of different datasets and algorithms.

To address these challenges, future research directions include employing diverse data augmentation techniques to simulate varied imaging scenarios and, thus, enhance model robustness [108], adopting active learning and self-supervised strategies to significantly reduce dependency on manual annotations [109,110], and leveraging model compression [111] methods to facilitate fast, offline inference on mobile devices. Furthermore, federated learning could continuously improve algorithms while ensuring user privacy [112].

## 3. Discussion

Given the insidious and gradual nature of early oral disease symptoms, early detection and timely intervention are crucial to prevent disease progression. Current diagnostic methods, typically dependent on specialized equipment and clinical evaluations in hospitals, often lead to low consultation rates and missed early lesions. Extending diagnostic procedures to home-based, convenient, and routine self-screening can enable the earlier detection and timely management of oral disease risks, significantly improving oral health outcomes.

Due to the frequent occurrence of dental diseases at multiple sites within the dentition and their concealed nature, the key to efficient screening and precise treatment lies in the simultaneous monitoring of multiple intraoral sites across the entire dentition. However, current screening technologies often fail to precisely locate lesions, primarily providing results limited to either single-site or general oral cavity assessments (Table 2). Furthermore, these methods typically require specialized equipment and skilled operations, limiting the patient’s compliance for routine home use. Accuracy can also be compromised by variations in biomarker specificity, individual biological differences, and environmental factors. Additionally, the high costs, potential declines in sensitivity over time, and calibration drift further challenge their compliance with FDA regulatory standards [113].

In comparison, AI-assisted visualized distributed biochemical detection methods are convenient, reliable, and do not require specialized equipment, presenting significant potential for home-based dental disease screening. However, several challenges remain before widespread application, including the establishment of standardized evaluation criteria for AI diagnostic models, the enhancement of dataset diversity and model robustness through data augmentation techniques, rapid offline inference via model compression methods, and continuous algorithm optimization with federated learning to protect user privacy. With continued progress in these areas, AI-assisted result interpretation is expected to revolutionize home-based oral disease management by enabling simple yet precise biochemical detection.

## 4. Opportunities and Perspectives

With the improvement of economic levels and the acceleration of the aging population, the demand for oral healthcare services is expected to rise significantly [114]. Concurrently, research linking oral diseases to overall health will continue to strengthen, fostering a broader consensus on the importance of oral health. This growing awareness will drive the development of convenient oral biochemical testing technologies. The future of these technologies depends on precise sampling, efficient signal amplification, and straightforward, equipment-free result interpretation.

A promising avenue for biochemical testing in oral disease detection lies in the study of metabolites from the oral microbiome. The oral cavity hosts over 700 microbial species, many linked to oral diseases and systemic conditions through circulation or ingestion [115]. Biochemical technologies that detect metabolites from the oral microbiome could provide critical insights not only into oral health but also into the development of systemic diseases.

Furthermore, combining wearable biochemical sensors with AI will advance the detection from merely identifying disease presence to accurately mapping lesion locations. This intelligent approach will expand oral health management, enabling individuals to self-monitor oral conditions. Moreover, oral wearable sensors hold considerable promise in monitoring chronic metabolic diseases like diabetes, gout, and hyperlipidemia. Multi-disease biochemical detection technologies using oral wearable sensors are expected to become a powerful tool in personal health management, offering precise and efficient monitoring that can improve the outcomes of overall health. However, several technical barriers must be addressed before such visions can be fully realized. These include signal interference arising from wireless noise or environmental factors [116], potentially compromising data quality and the accuracy of AI-based diagnosis; challenges related to device durability [117], especially the ability of sensors and wearable technologies to reliably operate under varying environmental conditions, such as temperature fluctuations and humidity; and significant concerns regarding data security and patient privacy [118], which necessitate robust measures to safeguard sensitive health data during transmission, storage, and analysis. Addressing these issues will require advancements in hardware design, algorithm optimization, and stringent cybersecurity protocols.

With ongoing research and innovation, biochemical detection technologies for oral disease risk warning are poised for unprecedented growth and application. In the near future, an integrated, efficient, and user-friendly oral health-monitoring system is likely to emerge, providing comprehensive protection for oral health, precision dentistry, and advancing the overall landscape of personal healthcare.

## Figures and Tables

**Table 1:** Biomarkers of major oral diseases at early stage.

Disease	Lesion Location	Pathological Process	Biomarkers
Dental caries	Teeth (enamel, dentin, pulp, etc.)	Microorganisms adhering to the tooth surface metabolize sugars from food, producing organic acids. These acids lead to the dissolution of minerals, such as calcium and phosphorus, from the enamel, resulting in its demineralization.	pH [28], lactate [29], acetate [30], Ca^2+^ [31], H_2_O_2_ [32], *Streptococcus mutans* (*S. mutans*) [33], and *Lactobacillus* [34]
Periodontitis	Periodontal supporting tissues (gingiva, periodontal ligament, alveolar bone, cementum, etc.)	Microorganisms in dental plaque proliferate, producing toxins and enzymes that trigger inflammatory responses in gingival tissue. Then, the inflammatory mediators cause both vasodilation and increased vascular permeability. Immune cells, including neutrophils and lymphocytes, migrate to the lesion site, releasing pro-inflammatory factors and enzymes.	H_2_S [35], TNF-α [36], IL-1β [37], MMP-8 [38], human neutrophil hydrolase (HNE) [39], cathepsin-G [40], gingipain [41], β-glucuronidase [42], typsin-like enzyme [43], PI3K [44], *Porphyromonas gingivalis* (*P. gingivalis*) [45], and *Actinobacillus actinomycetemcomitans* [46]
Oral cancer	Oral mucosa (tongue, buccal mucosa, gums, floor of mouth, hard palate, soft palate, etc.)	Abnormal changes, such as leukoplakia, erythema, and lichen planus, can present in the oral mucosa. In these regions, epithelial cells proliferate abnormally, leading to morphological changes, such as enlarged nuclei, prominent nucleoli, and disrupted cell polarity.	CYFRA-21-1 [47], IL-8 [48], fibrin [49], and YAP1 [50], HPV DNA [51], HPV E6 antibody [52]

**Table 2:** Summary of key performance indicators for the as-discussed technologies.

Sample Resources	Sensor	Limit of Detection (LOD)	Sensitivity	Selectiveness	Cost	Ease of Use
Exhaled Breath	Au/In_2_O_3_-MPTES [58]	10 ppb H_2_S	At 10 ppm H_2_S, R_a_/R_g_ = 1505.3	High	High	Low
	Manganese Oxide-Nanosheets [68]	20 ppb MM	40 Hz/1000 ppb	Middle	High	Low
	Cu_2_O/ZnO heterojunctions [65]	10 ppb H_2_S	At 0.01 ppm H_2_S, I_g_/I_a_ = 62	High	High	Middle
	Co-MOFs heterojunctions [59]	1.3 ppb H_2_S	At 10 ppm H_2_S, R_a_/R_g_ = 32	Middle	High	Middle
	Fe-MoO_3-x_/TiO_2_ [69]	0.34 ppb H_2_S	At 0.2 ppm H_2_S, R_a_/R_g_ = 4.9	Middle	High	Middle
	BISS-PAAm based structural color hydrogel [60]	61 ppb VSCs	177.87 nm/mM	Middle	Middle	High
	Cu-TATB@paper [23]	8 nM VSCs	148 a.u./μM	High	Middle	High
Whole Saliva	Anthocyanins-based paper points [79]	/	−20 ± 0.5	Middle	Low	High
	MNPs [81]	49 CFU/mL *P. gingivalis*	/	High	Middle	Middle
	MNPs [80]	1 pg/mL HNE, 100 fg/mL Cathepsin-G	/	High	Middle	Middle
	RPA-LF strip [87]	6.40 × 10^−4^ μg/mL *P. gingivalis* DNA	/	High	Middle	Middle
	Fe_3_O_4_ NPs-based DNA-Nanozyme Interfaces [24]	12 CFU/mL *S. mutans*	/	High	Middle	Middle
	PMMA-based Aptasensor [89]	0.011 nM ODAM	3854.2/nM	High	High	Low
Local Exudate from Si-ngle Site	Bifunctional graphene [25]	single bacterium	At 100 CFU/mL bacterium, ∆R/R_0_ = −0.05	High	Middle	Middle
	PANi-based dental patch [99]	/	−62.97 mV/pH	Middle	Middle	Middle
	Modified PNIPAM hydrogel RF-Trilayer Sensor [100]	/	6 MHz/1000 (mg/dL)	Middle	Middle	Middle
	AG-AgNPs-CHL hydrogel RF sensor [101]	1.2 μM H_2_S	0.62/μM	High	Middle	Middle
Local Exudate from the Whole Dentition	Nitrazine-Yellow based dental floss [79]	/	−33.8 ± 1.5	Middle	Low	High
	ZnO-PDMS Mouthguard [102]	/	/	High	Low	High
	Au@Ag Nanorods-PDMS Mouthguard [26]	7.86 ppm H_2_S	0.34/ppm	High	Low	High
	ZnO QDs-PDMS dental patches [103]	20.3 μM H_2_S	−1255.4 a.u./μM	High	Low	High
